# Evolution of Bird and Insect Flower Traits in *Fritillaria* L. (Liliaceae)

**DOI:** 10.3389/fpls.2021.656783

**Published:** 2021-03-31

**Authors:** Katarzyna Roguz, Laurence Hill, Agata Roguz, Marcin Zych

**Affiliations:** ^1^Botanic Garden, Faculty of Biology, University of Warsaw, Warsaw, Poland; ^2^Petersham Lodge, Richmond, United Kingdom; ^3^Feature Forest, Gdańk, Poland

**Keywords:** pollinator shift, nectar, ancestral state, flower colour, floral syndrome, diversification, ornithophily

## Abstract

Pollinators are often perceived as a primary selective agent influencing flower traits such as colour, size, and nectar properties. The genus *Fritillaria* L. (Liliaceae), comprising approximately 150 species, is described as generally insect pollinated. However, there are at least three exceptions: two hummingbird-pollinated North American species and one passerine-pollinated Asian species. Despite this variation in pollination, little is known about flower traits that may accompany this shift in fritillaries. In this study, we aimed to assess the attractiveness of the floral traits for (new) pollinators and track the evolution of flowers traits in the context of a shift in the principal pollinator. Therefore, we studied 14 flower traits related to the pollination in 60 *Fritillaria* species and traced the evolutionary trajectory of these traits. We used a phylogenetic tree of the genus, based on five DNA markers (*matK, rpl16*, and *rbcL*, 18S, and ITS) to reconstruct the ancestral state of studied flower traits. The results show that in bird-pollinated species several new traits evolved. For example, flower colouration, nectar sugar, and amino acid concentration and composition fulfil the criteria of ornithophilous flowers, although flower traits do not exclude insect pollinators in bird-pollinated fritillaries. Interestingly, we recorded potential reversals from bird to insect pollination. Our analysis, showing a broad study of flower traits among closely related species in the context of pollinator shift, serves as a starting point for future work exploring the genetic and physiological mechanisms controlling flower traits in the genus *Fritillaria.*

## Introduction

Angiosperms present enormous variation in flower traits, such as colour, shape, size, content and quality of the floral reward. This variation is also encountered among closely related species (e.g., Johnson et al., 1998; Wilson et al., 2004; Smith et al., 2007; Thomson and Wilson, 2008; Guzman et al., 2017; Roguz et al., 2018). Since Darwin (1862), flower divergence has largely been attributed to the selection of pollinators. The phenomenon of adaptation to the preferences of the most common and efficient pollinator has been formalised as pollination syndromes—recurring suites of flower traits associated with different groups of pollinators (Faegri and van der Pijl, 1966). Despite the ongoing debate about the assumptions of this concept (Ollerton, 1996; Waser et al., 1996; Rosas-Guerrero et al., 2014; Dellinger, 2020) it serves as a framework for studies of floral diversity (Kay and Schemske, 2003; Smith et al., 2007).

Several empirical studies have supported the relation between flower traits and pollinators groups (Dellinger, 2020). This relation is even more visible, when pollinators shifts are considered. These new pollinators may be more efficient (Ashworth et al., 2015), or the only present, if e.g., the occurrence of pollinator shift is enforced by the loss of ancestral pollinators (Cox and Elmqvist, 2000). Indeed, significant changes in floral traits, such as corolla symmetry, petal colour, and type of reward, are generally associated with pollinator shifts, strongly supporting the idea that flower traits reflect new pollinators preferences (Bruneau, 1997; Ramírez-Aguirre et al., 2019; Barrionuevo et al., 2021).

Among visual flower traits, colour is one of the key features involved in signalling to pollinators (Willmer, 2007). Colour constitutes to be one of the main traits used in floral syndromes (Faegri and van der Pijl, 1966), and although its attractiveness for specific pollinators groups remains a source of contention, the assumption that e.g., bird pollinated flowers are red remains common (Smith et al., 2007). In *Penstemon* Schmidel, for example, red petal colour was one of the characters that best predicted hummingbird visitation (Wilson et al., 2004). Flower colour change was also an important step involved in the transition from bee to nocturnal hawk moth pollination in the genus *Petunia* Juss (Hoballah et al., 2007). Flower colour, however, does not always relate well to pollinator shifts (Smith et al., 2007; Opedal, 2019; Roguz et al., 2020a). For example, red colouration may be a good predictor of bird-pollination in *Iris* L. species, but not vice-versa (Roguz et al., 2020a). The explanation for this lack of correlation is that ultimate flower choice is based on a combination of stimuli. Moreover, most flower species are pollinated by generalists (Reverté et al., 2016).

Other signal-transmitting flower traits, such as flower size and the arrangement of its reproductive parts, can also be subjected to strong selection pressure (Lavi and Sapir, 2015; Souto-Vilarós et al., 2018). For more efficient pollen transfer, flower size and arrangement of reproductive parts should fit the body and behaviour of the pollinator (Fenster et al., 2006). Studies conducted on *Ipomopsis* Michx. have shown that the display size was positively correlated with hummingbird importance and negatively correlated with dipteran importance (Smith et al., 2007). However, like in the case of colour, shift to the new pollinator does not always result in a consistent flower size or the arrangement of flower reproductive parts (Dupont et al., 2004).

While visual flower traits are an important signal for potential pollinators, floral rewards are also crucial in shaping the interaction. In most cases plants provide a food reward of either pollen or nectar (Parachnowitsch et al., 2018). In the latter case, animals visiting flowers may also exert selection on floral reward properties, such as volume, sugar, or amino acid (AA) concentration (Dupont et al., 2004). Flower reward chemistry was proposed as a key factor to transition from moth to bee pollination in *Satyrium* Sw. orchid (Castañeda-Zárate et al., 2021). Also in the case of *Ipomopsis* reward was positively correlated with hummingbirds importance. However, in *Clivia* Lindl. the shift from bird- to butterfly pollination was accompanied by the change of flower shape, smaller nectar volume, and emission of scent, while flower colour and nectar chemistry were not substantially modified (Kiepiel and Johnson, 2014).

The set of new flower traits depends on the identity of the new pollinator. The most often recorded pollinator shifts include the transition from insect (bee)-pollinated to bird-pollinated plants (Wilson et al., 2006; Thomson and Wilson, 2008), which is usually caused by the higher efficiency of birds in transferring pollen as well as lower pollen discounting (Thomson and Wilson, 2008). For example, in *Penstemon* and related genera, there have been at least 10 separate transitions toward hummingbird pollination (Wilson et al., 2007). In *Mimulus* sect. *Erythranthe* hummingbird pollination has arisen from melittophily twice (Beardsley et al., 2003). The following genera also include hummingbird-pollinated plants and species that are pollinated by other bird lineages: *Agave*, which is predominately bat-pollinated (Agavaceae, Good-Avila et al., 2006); *Erythrina*, which is entirely ornithogamous (Fabaceae; Bruneau, 1997; Etcheverry et al., 2012), *Mucuna* (Fabaceae; Kalin Arroyo and Penaloza, 1990), and *Puya* (Bromeliaceae, Hornung-Leoni and Sosa, 2005).

The above examples and other pollinator shift studies show that floral evolution has been highly labile and also directional. Moreover, even specialised pollination systems are not a dead-end (Tripp and Manos, 2008). There have been few reverse transitions from bird to insect pollination (Mast et al., 2012); for example, in the tribe *Sinningieae* (Gesneriaceae; two transitions from hummingbird to bee pollination, Perret et al., 2003) and in the genus *Aquilegia* (Ranunculaceae; five transitions from hummingbird to hawk moth pollination, Whittall and Hodges, 2007).

Such shifts to new pollinators, or reverse transitions to an ancestral pollinator, usually result in a different suite of traits, attracting new, more efficient pollinators and repelling less effective pollinators or flower robbers (Castellanos et al., 2004; Salas-Arcos et al., 2017). While flower colour-related traits, which often occur with pollinator shift, are well understood, little is known about the other flower traits, like nectar properties, that are the potential outcome of this shift. To understand the influence of the new pollinators on flower traits, it is necessary to explore plant genera exhibiting pollinator shift.

In our study, we focussed on *Fritillaria* L. (Liliaceae), which is a genus that includes approximately 150 species of bulbous plants, predominantly found in temperate Holarctic regions of both the Old and New World (Tamura, 1998; Day et al., 2014; Zych et al., 2014). The highest diversity of *Fritillaria* is observed in the Mediterranean region (Beetle, 1944; Rix, 1984; Zaharof, 1986; Rønsted et al., 2005; Tekñen and Aytaç, 2011; Hill, 2016; Kiani et al., 2017). *Fritillaria* flowers are generally actinomorphic and have a nodding tulip-like trimerous perianth. Despite the similarity, flowers of fritillaries exist in various sizes and colours, and can be white, pink, greenish, yellow, or purplish/reddish. We still do not fully understand the diversity of fritillaries, but it is possibly related to pollination systems, at least to some extent. Most fritillaries are described as or presumed to be pollinated by insects; however, there have been at least two pollination shifts from insect to bird pollination in the genus. This shift involves distinct bird groups, namely passerines and hummingbirds (Búrquez, 1989; Peters et al., 1995; Pendergrass and Robinson, 2005) and results in new flower traits in fritillaries. For example the only red and orange fritillaries are pollinated by birds. Moreover, properties of nectar reward reflect preferences of their bird pollinators. Hummingbird-pollinated fritillaries produce nectar of medium concentration and with medium AA concentration, while passerine bird pollinated species produce huge amounts of AA rich nectar, dominated by sucrose preferred by passerines. Still, most of the members of this genus, which are presumably insect pollinated, produce smaller amounts of balanced nectar (Roguz et al., 2018, 2019). Also within insect pollinated fritillaries, there may have occurred shifts of the main pollinators. *Fritillaria camtschatcensis* is a species with distinct flower traits and reward properties (Roguz et al., 2018, 2019), pollinated by flies (Zox and Gold, 2008). Nectaries of this species are covered with protrusions. Traces of hardly accessible, viscous and almost solid nectar are available as a thin film overlying the protrusions (Roguz et al., 2018, 2019). This way of nectar presentation likely excluded insects other than flies with a cushion-like labium.

*Fritillaria* is also a genus in which reversals from bird to insect pollination may have occurred. *Fritillaria raddeana* is a species closely related to passerine bird pollinated *F. imperialis* (Wietsma et al., 2015). However, flowers of *F. raddeana* are greenish, smaller and this species produces small amounts of highly concentrated, balanced nectar (Roguz et al., 2018, 2019). The question remains, what was the characteristic of the common ancestor of this species.

Given the unique pollination system (at least two transitions from insect to bird pollination, possible reversals), our research goals were to build a phylogenetic framework in which flower traits and reward properties within *Fritillaria* can be evaluated. We asked the following questions: to what extent do flower traits fulfil criteria of ornithophilous/enthomogamus flowers? Can quantitative traits, for example flower size, influence the diversification dynamic? Do shifts in phenotypic optima of flower traits overlap with the pollinator shift? We tested the assumption that nectar (as an important flower reward) and colour (as an important visual sign) play crucial roles in pollinator shits. We also anticipated reversals from hummingbird-bird to insect-pollination because of the low degree of specialisation of bird-pollinated fritillaries.

## Materials and Methods

### Phylogenetic Tree

The first step in the analysis of flower trait evolution in fritillaries was to create a phylogenetic tree of the genus. In this study, we prepared a database of five DNA markers: plastid genome (*matK*, *rpl16*, and *rbcL*), nuclear (18S), and internal transcribed spacer (ITS) sequences. These genetic markers have been used successfully to infer phylogenetic relationships in *Fritillaria* (Rønsted et al., 2005; Day et al., 2014; Khourang et al., 2014; Kim and Kim, 2018). We used *Lilium* L. as an outgroup based on the established relationship between *Fritillaria* and this genus (Rønsted et al., 2005; Day et al., 2014). We selected 11 *Lilium* species with the highest gene coverage (accession numbers in Supplementary Material 1).

The *matK* region of two *Fritillaria* species, *F. biflora* and *F. olgae*, was sequenced by extracting DNA from the collection of living plants at the University of Warsaw Botanic Garden (BG). We tried to obtain sequences of different regions, but due to methodological difficulties, only those for the *matK* regions were achieved (methods description Supplementary Material 2). All but two sequences used in the present study were acquired from GenBank. The sequences were downloaded using the MatPhylobi program (Kranas et al., 2018), which is a command-line tool for constructing taxonomic data sets for phylogenetic inference based on NCBI data. To create the sequence database in MatPhylobi, we selected *F. michailovski* and *L. regale* as representatives of the studied genera and used them as species to construct the data set. Overall, taxon sampling for *Fritillaria* totalled 461 accessions (Supplementary Material 1). We prepared two databases with the downloaded sequences. One database was prepared to analyse 60 *Fritillaria* species, for which we were able to obtain information about flower traits, flower reward properties, and pollinators. The second database was prepared for the flower colour analysis, in which we surveyed 119 species. This analysis was conducted for all species where we were able to obtain both the information about colour and the sequences.

Both databases were prepared following the same procedure. After downloading with MatPhylobi, all five markers were independently aligned using the multiple alignment program MAFFT (version 7; Katoh et al., 2017; method = “localpair” incorporating local pairwise alignment information, maxiterate = 1,000). Then, all alignments were imported into Mesquite for visual inspection (version 3.6; Maddison and Maddison, 2018). Poorly aligned positions and regions with high gap density in each alignment were eliminated using the Gblocks program (Version 0.91b, Castresana, 2000; Talavera and Castresana, 2007). The trimmed alignments were then concatenated with catfasta2phyml into a single combined alignment^1^. For each locus, we selected the appropriate evolutionary model recommended by the ModelTest-NG (version 0.1.3; Darriba et al., 2020). Selected models were as follows: matK:GTR + I + G4, rpl16:GTR + I + G4, rbcL:TVM + I + G4, 18S:TrN + G4, ITS:GTR + G4.

The obtained alignment was used in RAxML to generate phylogenetic trees. RAxML was used for a maximum-likelihood (ML) analysis (version 8.0; Stamatakis, 2014). Bootstrap (hereafter bsp) analysis with 1000 replicates was conducted on each partition.

### Studied Characters: Flower Traits, Reward Properties, and Pollination System

The next step was to prepare a database describing the diversity of *Fritillaria* flower traits, the divergence of floral reward offered for pollinators, and the pollination system. Since most *Fritillaria* species are rare and grow in poorly accessible places, collecting data in natural habitats, although crucial, is not possible for most fritillaries. Nonetheless, to gain insight into the evolution of members of this genus, we used flowers from plants cultivated in University of Warsaw Botanic Garden and in the private collections of Colin Everett (Somerton, Somerset, United Kingdom; hereafter CE), one of the co-authors Laurence Hill (Richmond, Surrey, United Kingdom; hereafter LH), and Paweł Kalinowski (Szczeglacin, Korczew, Poland; hereafter PK). Most *Fritillaria* species are also rare in cultivation; hence, the number of specimens used for each type of analysis varied due to the availability of fresh plant material (the accession numbers and sources of plant material are listed in Table 1).

**TABLE 1:** Investigated *Fritillaria* species, all flower measurements in millimetres ± standard deviation, values in () indicate number of measurements, ^†^ indicates values obtained from literature.

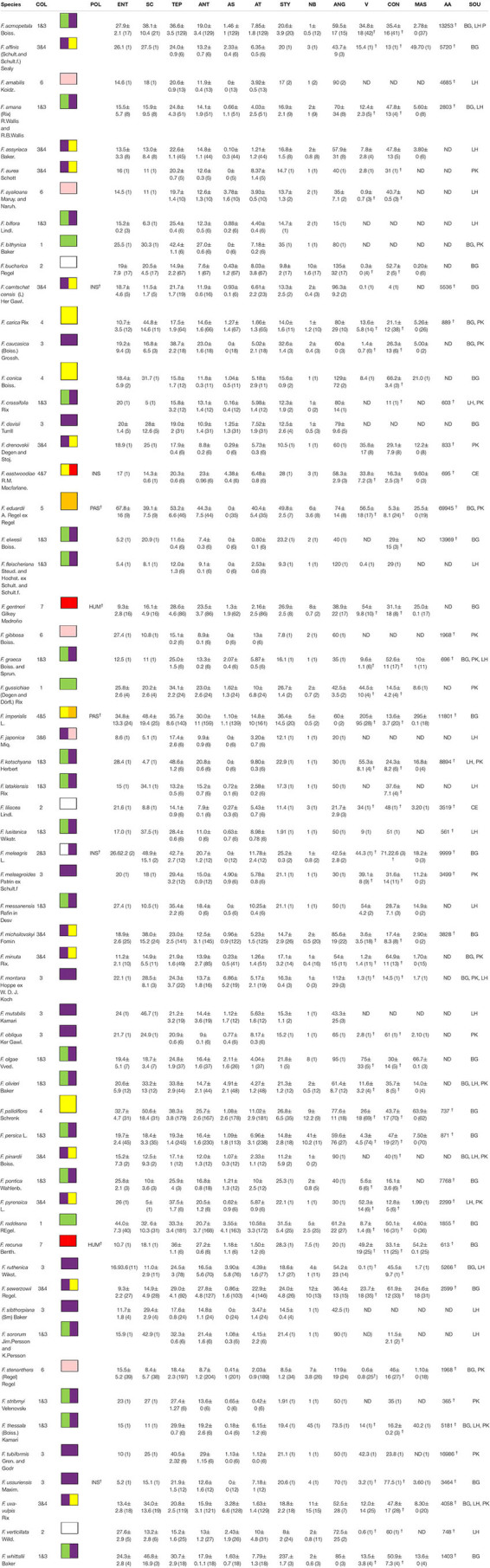

**COL—flower colour (1—green, 2—white, 3—purple, 4—yellow, 5—orange, 6—pink, 7—red), POL—main pollinator (INS—insects, HUM—hummingbirds, PAS—passerine birds), ENT—entrance diameter, SC—scape length, TEP—tepal length, ANT—stamen length, AS—anthers-style distance, AT—anthers-tepals distance, STY—style length, NB—number of flowers in the inflorescence, ANG—angle between the stem and the middle of the flower, V—nectar volume [μL], CON—nectar sugar concentration [%], MAS—nectar mas [mg], AA—total amount of amino acids [pmol/μL], SOU—source of obtained plants.**

**FIGURE 1:**
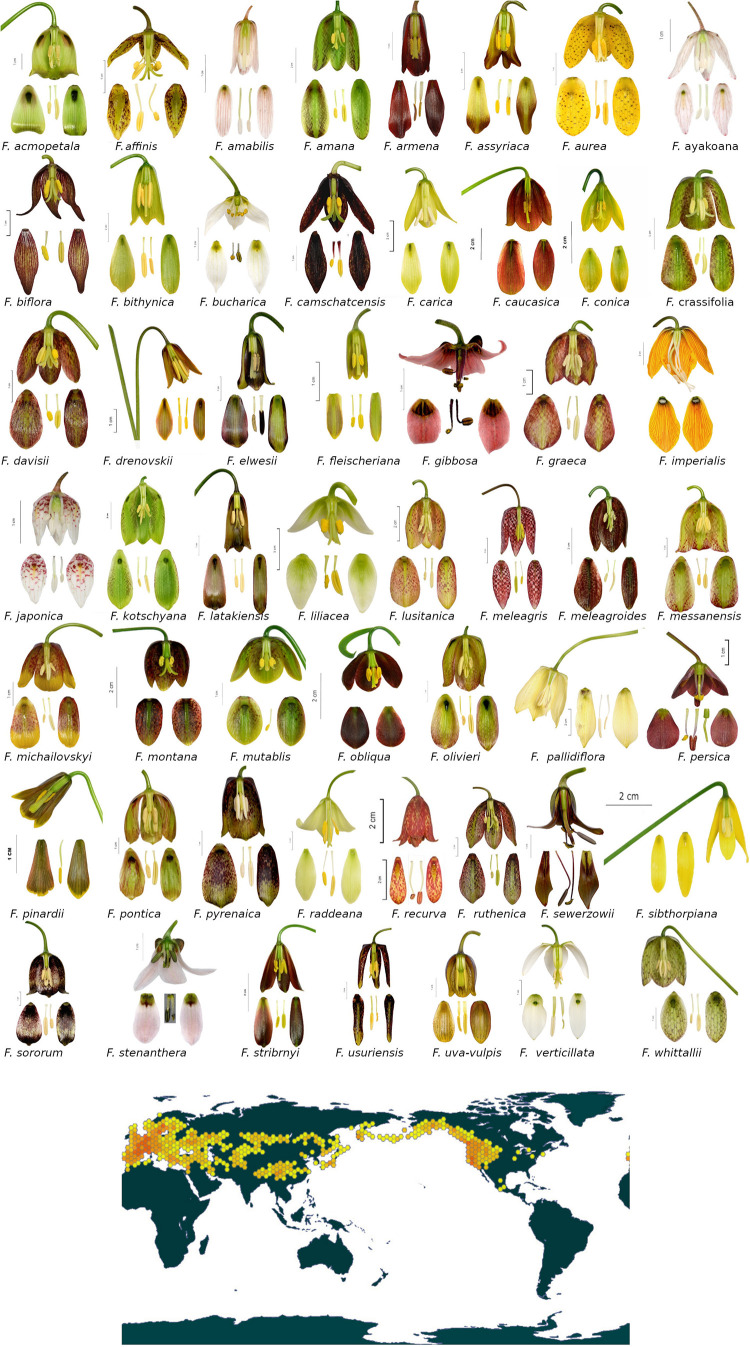
Flowers of some *Fritillaria* species presented in the study (pictures LH); map showing distribution of Fritillaria species (source: Fritillaria L. in GBIF Secretariat (2019). GBIF Backbone Taxonomy. Checklist dataset https://doi.org/10.15468/39omei accessed via GBIF.org on 2021-02-24).

In the case of flower colour, which is one of the most important traits shaping plant–pollinator interaction, we were not restricted by the availability of plant material. Flower colour was assessed for all 119 species, as we were able to score the colour without plant material (source of information^2^ ^,3^,^4^). Flower colour was scored by one of the authors, and the most dominant colouration of inner and outer side of the tepals was noted (e.g., we did not consider greenish colouration of the base of the tepals). Fritillary flowers exist in various colours (Figure 1). For the present study, we used simple colour-categories based on flower colour as perceived by humans. Although UV reflecting flower parts may be an important part of petals colouration, therefore shaping plant-pollinator interaction, we were not able to do a genus-level analysis due to a lack of relevant data. The availability of petal reflectance data is limited among fritillaries. In the present study flowers were categorised as green, orange (intermediate tint between yellow and red), pink (intermediate tint between red and white), purple (intermediate tint between blue and violet), red, yellow, or white. In the case of *Fritillaria* flowers representing similar colour groups e.g., pink and purple the visible differences are unambiguous. Species exhibiting colour polymorphism (e.g., *F. persica* and *F. imperialis*), or with bi-coloured flowers (e.g., *F. michailovskyi*) were coded into multiple categories.

Fourteen traits for 60 *Fritillaria* species were included in the floral trait analysis, which were broadly divided into two categories: flower and reward traits (Table 1). We hypothesised that these traits are subject to natural selection by pollinators. To estimate the potential attractiveness of the flower, we assessed the display size (number of flowers per plant). We also assessed tepal length as a measure of flower size (at its longest point; scheme showing the described measurements Supplementary Figure 1). To assess the fit between the pollinators and the flower reproductive parts, we measured the stamen and style length (from the base of the flower to the apex of the studied element), the distance between the anthers and the tepals and the anthers and the style, respectively (measured between the anthers tips and the tepals/style). Flower accessibility may play a crucial role in shaping plant–pollinator interactions; therefore, we recorded the orientation of flowers on the stem (the angle between the stem and the middle of the flower), the length of the scape, and the diameter of the corolla entrance (measured along the stem axis). All measurements were conducted using a digital calliper, Borletti DIN 862 (Borletti, Italy), which was connected to a computer to automatically record the values.

Because of the importance of floral reward for pollinators, we also included nectar properties; that is, volume, mass, sugar and AA concentration. Data on nectar AA concentration for all species, and nectar sugar concentration and volume for 45 species were derived from the literature (Roguz et al., 2018, 2019). In this study we acquired nectar data for *F. assyriaca, F. aurea, F. conica, F. drenovskii, F. japonica, F. fleischeriana, F. messanensis*, and *F. verticillata* (Table 1). Nectar was sampled, and the volume and sugar concentration were analysed as described by Roguz et al. (2018).

Finally, we collected data on the pollination system of the fritillaries. Pollinators were determined based on the literature (White, 1789; Búrquez, 1989; Peters et al., 1995; Pendergrass and Robinson, 2005; Zox and Gold, 2008; Zhang et al., 2010; Zych et al., 2014; Guo et al., 2017), in which flower visitors were assessed through direct observations.

### Phylogenetic Studies

The obtained *Fritillaria* tree and trait database were used to reconstruct the ancestral state of studied flower traits and floral reward properties. The ancestral states were inferred on an ultrametric tree, generated using the *chronos* function in the “ape” package (with the age of the tree set to one, value of smoothing parameter lambda = 0 based on log-likelihoods Paradis et al., 2004). For each trait, we first determined the appropriate transition probability model. Transitions among all possible states may occur at the same rate, but it is supposed that it is easier to lose a complex character than to gain one. To include possibility for such asymmetries in rates we determined the transition probabilities using a log-likelihood ratio analysis, choosing from: ER (equal rate), SYM (symmetrical rate), and ARD (all-rates different). The ARD transition ratio model was chosen in all cases because it had the highest likelihood value (*make.simmap* function in the “phytools” package, Revell, 2012).

To infer the ancestral states of one polymorphic flower trait in our database, that is, flower colour, we used maximum likelihood estimation of the rates to determine the state probabilities (*rayDISC* function—this function specifically accommodates characters with polymorphic states; “corHMM” package; Beaulieu et al., 2013). To reconstruct the ancestral states of the continuous traits (e.g., tepals length or nectar volume), we used maximum likelihood estimation for the continuous traits (*FastAnc* function; “phytools” package; Revell, 2012).

Since pollinator shifts are often accompanied by changes in flower traits, we estimated the shifts in phenotypic trait optima of the studied traits. To do so, we used a least absolute shrinkage and selection operator (LASSO) procedure as proposed by Khabbazian et al. (2016) (*estimate_shift_configuration* function in the “l1ou” package) and phylogenetic-aware information criterion (pBIC) to perform model selection. We chose to use the phylogenetic pBIC for model selection as it minimises the inference of false shifts (Khabbazian et al., 2016). This method detects past changes in the expected means of the trait values using the Ornstein-Uhlenbeck process. The number of shifts in phenotypic trait optima was selected with the use of the Akaike information criterion (AIC, Ingram et al., 2013). Due to missing data in the reward properties, shifts in phenotypic trait optima were calculated for two groups. In the first variant, shifts were calculated on the basis of flower traits (except for anther-style distance where too many 0 values hampered the analysis) and in the second, shifts were calculated only on the basis of reward properties.

Flowers play an important role in shaping plant–pollinator interactions, and consequently, in plant reproduction. Therefore, floral traits have been hypothesised to influence the diversification dynamics of plant lineages. To test this, we performed a trait-dependent diversification analysis. Some studies have shown that for quantitative traits, state-dependent speciation-extinction models (e.g., Quantitative State Speciation and Extinction) may result in elevated false discovery rates. Therefore, in our study, for the quantitative traits we used an alternative trait-dependent diversification method, which does not model the relationship between traits, but tests for correlations between summary statistics of phylogenetic branching patterns and trait variation at the tips of a phylogenetic tree (*ES-sim* function, Harvey et al., 2017). The test assumptions were the same as for fitting a Brownian motion model to phylogenetic comparative data.

Finally, we assessed if there is a tendency for closely related *Fritillaria* species to resemble one another more than distantly related ones, that is, if there is a phylogenetic signal. To assess the strength of the phylogenetic signal on continuous data, we applied Blomberg’s *K* (Blomberg et al., 2003) (*phylosig* function in the “phytools” package, Revell, 2012). Blomberg’s *K* is a scaled ratio of the variance among species over the contrast variance. To incorporate estimation error, we used within-species variance. Species with a single observation and missing values were excluded from the analysis.

It is important to note that our study had inherent bias. Several studies have shown that *Fritillaria* diversity is centred on East Asia (Day et al., 2014; Huang et al., 2018; Kim and Kim, 2018; Li et al., 2018), the area from which the Liliaceae family originated (Huang et al., 2018; Kim and Kim, 2018). Unfortunately, this study included little material from SW China; therefore, any hypothesis of the ancestral form could change if material from this region was included. We also did not include hybridisation, which probably played an important role in the evolution of the studied genus (Zaharof, 1986), causing some incongruence in the phylogenetic analysis (Rønsted et al., 2005). This is because hybridisation has a small impact on the ancestral state reconstruction and on the parameter estimation (Bastide et al., 2018).

## Results

### Phylogenetic Tree

**FIGURE 2:**
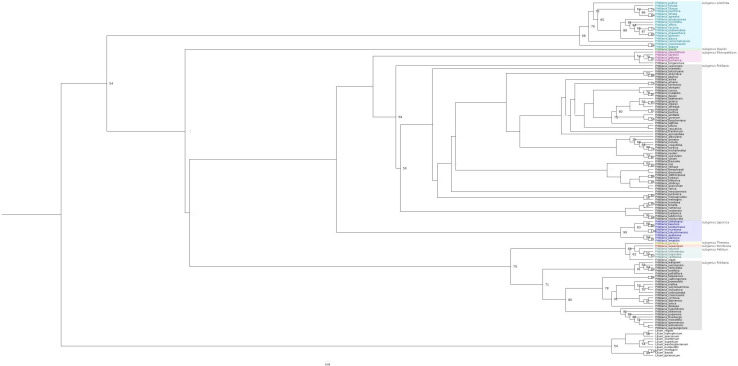
Maximum likelihood tree inferred from analysis combined of five DNA markers: plastid genome (*matK, rpl16*, and *rbcL*), nuclear (18S), and internal transcribed spacer (ITS) sequences. The bootstrap values are given along the branches (only values > 50 presented). Species representing different subgenera marked with different colours. Species not classified to any subgenus are left uncoloured.

The phylogeny analysis included in our study covered approximately 76% of the species currently recognised in the *Fritillaria* genus (we discuss a phylogenetic tree obtained for all species sequences available in GenBank; Royal Botanic Gardens, Kew, 2020), with *matK* having the highest coverage (97%), and ITS and 18S having the lowest coverage (both 59%). The analysis resulted in a tree based on five sequences resolving the *Fritillaria* genus as monophyletic (the final tree presented on Figure 2 and contributing sub-trees are presented in Supplementary Figure 2). However, subtrees inferred from plastid sequence data do not resolve *Fritillaria* as monophyletic, with the *Lilium* species nested within *Fritillaria*. Our final phylogenetic tree showed one large, polyphyletic *Fritillaria* subgenus (containing approximately 100 species, bsp values 59 and 71, respectively), and several smaller subgenera: *Liliorhiza* (66 bsp), *Rhinopetilium* (52 bsp), *Japonica* (99 bsp), *and Petilium* (62 bsp). There were also three subgenera consisting of one species: *Korolkovia* (63 bsp), *Theresia* (79 bsp), and *Davidii* (54 bsp). The polyphyletic *Fritillaria* subgenus consists of two clades: one containing mainly European, Middle Eastern, and North African species (including some species with ranges extending to China), and the second containing mostly Asian species, forming a separate clade (Figure 2).

### Studied Characters: Flower Traits, Reward Properties, and Pollination System

Of the 119 *Fritillaria* species included in the colour analysis, 47.9% had flowers in shades of purple and 18.5% had flowers in shades of green. The rest was distributed among yellow, pink, orange, red, and white, with several species representing two categories. Seven species were categorised as having bi-coloured or polymorphic-coloured flowers (Supplementary Material 3). The only red and orange flowers were found among bird pollinated species.

Most of the *Fritillaria* species had only one flower (4.98 ± 8.25 [(hereafter mean ± SD], range 1–52); however, there were several species with a large flower display, including, *F. persica* with more than 50 flowers in the inflorescence. Species described as bird pollinated always had more than one flower. In most cases, the tepal length in *Fritillaria* ranged from 10 to 50 mm (26.8 ± 9.23 mm), except for the *Petilium* subgenus, which had comparatively large flowers (40.4. ± 8.27 mm). Tepals were usually longer than anthers (18.2 ± 35.2 mm, range 5–63 mm), and anthers were shorter than stigmas (18.5 ± 10.5, range 1.72–67 mm). The arrangement of the reproductive element was variable. The distance between the anthers and tepals was usually larger (8.02 ± 7.68 mm, range 0–57 mm) than that between the anthers and stigmas (3.19 ± 6.00 mm). In several dozen fritillaries, the anthers touched the stigma, ranging between 0 and 32.2 mm. Fritillaries often presented nodding flowers on a long stem (27.1 ± 17.3 mm, range 3.88–87.6 mm). The angle between flower diameter and stem was 70 ± 35° (range 15°–180°). In some species, nodding flowers were also accompanied by a narrow entrance (22.6 ± 13 mm), however, some *Fritillaria* flowers have a wide entrance (range 5.4–83 mm).

The properties of the reward offered for flower visitors varied. We found differences in sugar concentration (38.5 ± 20.2%), with a large difference between the lowest (3.25%) and the highest (78%) values. Similarly, in the case of AA concentration (8137 ± 16,229 pmol/μL), with the lowest and highest values being 220 and 79,840.62 pmol/μL, respectively. Differences in the amount of nectar produced were also prominent, with the lowest value being less than 1 μL and the highest being 390 μL (32.5 ± 52.7 μL). *Fritillaria* flowers produced an average of 33.7 ± 7.9 mg of nectar (range 0.1–480 mg, Table 1).

**FIGURE 3:**
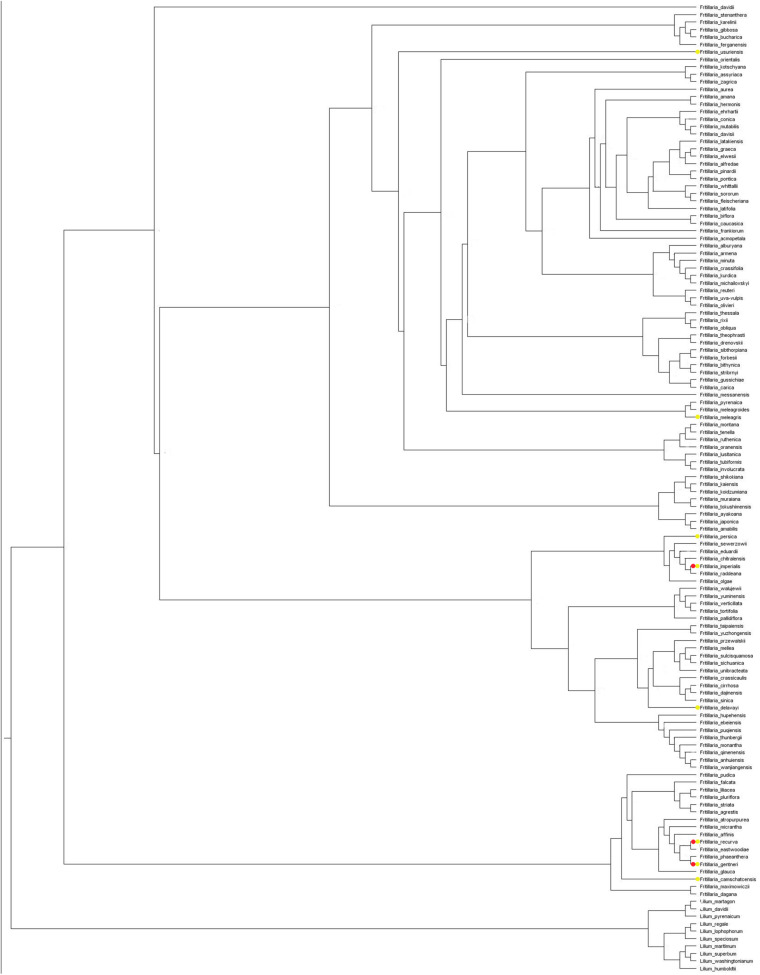
Maximum likelihood tree showing available information about pollinators of *Fritillaria* flowers (based on the literature data). Species marked with yellow dot are described as pollinated by insects, with red dot are described as pollinated by insects and birds. For species with no colour sign information about pollinators are not available.

Most of the *Fritillaria* species are described as pollinated by insects, with various bee species being the common observed visitors. However, there are several pollinator shifts noted. According to literature data, there is one shift to pollination by flies in the case of *F. camtschatcensis*. There are also at least two shifts toward ornithophily. Asian *F. imperialis* is pollinated by passerine birds, while North American *F. gentneri* and *F. recurva* are pollinated by hummingbirds (White, 1789; Búrquez, 1989; Peters et al., 1995; Pendergrass and Robinson, 2005; Zox and Gold, 2008; Zhang et al., 2010; Zych et al., 2014; Guo et al., 2017; Figure 3).

### Phylogenetic Studies

**FIGURE 4:**
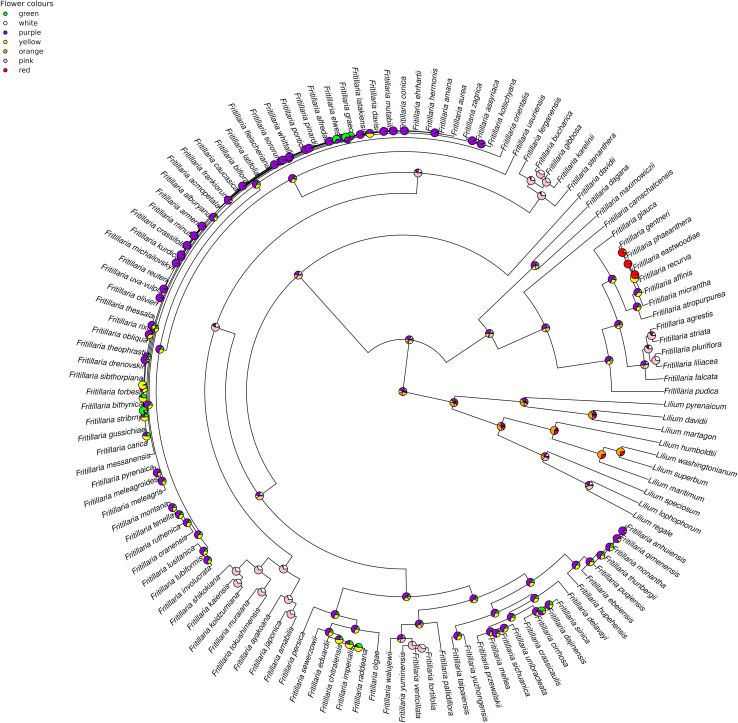
Estimation of ancestral states of flower colours among studied *Fritillaria* species calculated using maximum likelihood. Pie charts represent the proportion of the likeliest state at each internal node.

The ancestor of the fritillaries probably had purple or pink flowers. The internal nodes exhibit flowers with variable pigments, with purple colouration being the most common among both internal nodes and modern species. The results of our analysis indicate that several transitions were indeed reversals, for example, from yellow or green back to purple flowers (Figure 4).

The most recent common ancestor of fritillaries probably had more than one flower in the inflorescence, with the tepals, stamen, and styles of medium length (Supplementary Figure 3). In the case of subgenus *Japonica*, we observed a tendency for the size of the flower elements to decrease, while for the subgenus *Petilium* we noted the opposite tendency, that is, flowers and flower parts elongated in most species. Both the tepals and the stigmas were probably close to the anthers with variable tendencies in modern *Fritillaria* species (Supplementary Figure 4). The small distance between the reproductive elements in the most recent common ancestor may have also been related to the relatively small diameter of the nodding flowers, growing on a few centimetres-long scape (Supplementary Figure 5).

**FIGURE 5:**
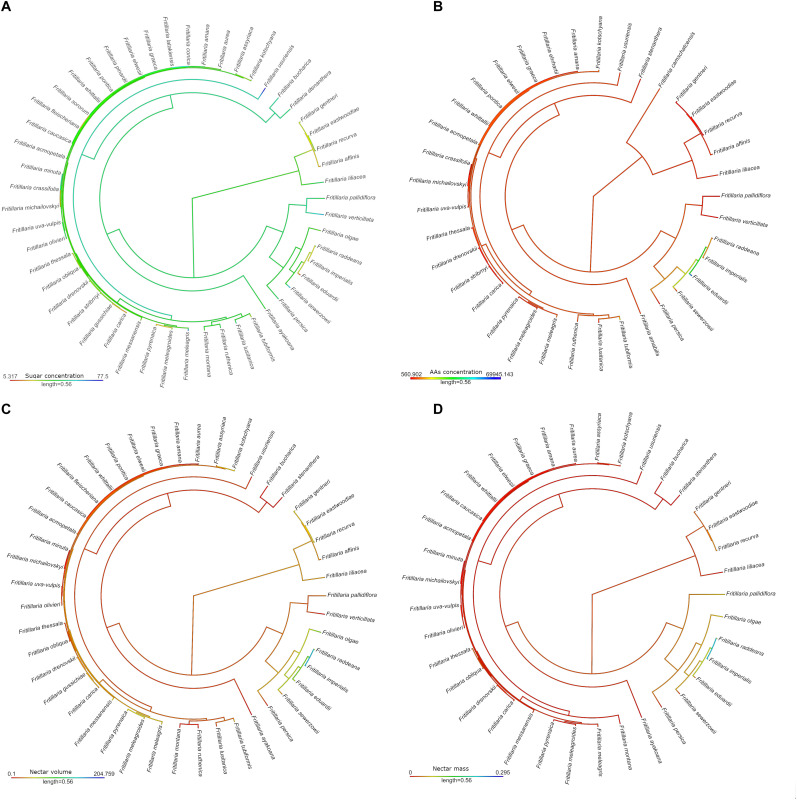
Maximum-likelihood ancestral state reconstruction for continuous traits among studied *Fritillaria* species: nectar sugar (%) **(A)** and amino acids concertation (pmol/μL) **(B)**, nectar volume (μL) **(C)** and mass (mg) **(D)**. Colour intensity shows the level of presented trait.

Ancestral state reconstruction of the characters related to the floral reward revealed that the ancestor probably produced a relatively small amount of nectar, which was rich in sugars but low in AAs (Figure 5). The most notable adaptations in flower reward properties were found among species representing the subgenus *Petilium*, e.g., large amounts of nectar with a low sugar concentration, but a high AA concentration.

**FIGURE 6:**
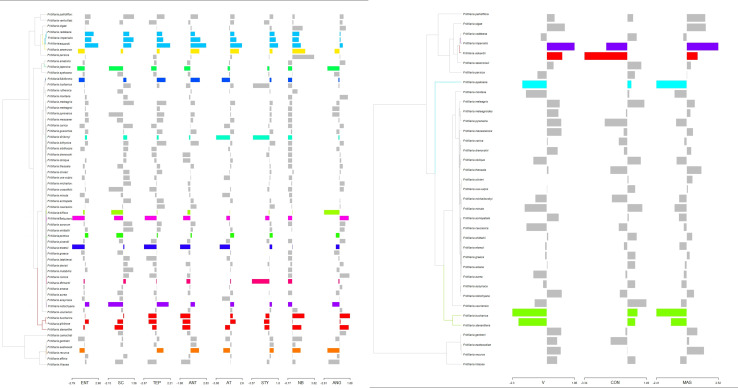
Estimated shifts in phenotypic optima of flower traits assessed for studied *Fritillaria* traits. Shifts in adaptive peaks are indicated with asterisks and convergent shifts in adaptive peaks have the same branch colour. Left: shift configuration. Right: bar graphs showing the traits combined for analysis. **(A)** Shifts calculated on the base of flower traits (with the exception of anthers-stigmas distance where too many 0 values hampered the analysis) and **(B)** reward properties. Difference in number of species in **(A,B)** results from missing data in the case of reward properties.

Using a LASSO procedure, we found support for 13 independent shifts in flower phenotypic trait optima and four shifts in the reward properties. For pollinator shift toward pollination by passerine birds both shift in phenotypic trait optima of flower traits and nectar properties coincide. Adaptation to pollination by new pollinators does not always coincide with the shift in the phenotypic optima of studied traits. For the species pollinated by hummingbirds we found shifts in phenotypic trait optima only for flower traits of *F. recurva*, while there were no shifts in the reward properties. We also did not notice such a shift in phenotypic trait optima in the case of e.g., *F. camtschatcensis*, a species pollinated by flies (Figure 6).

The Es-sim tests, which search for the relationship between change in continuous traits and diversification, showed no correlation between the summary statistics of phylogenetic branching patterns and studied traits. We found a statistically significant, although very weak, phylogenetic signal for Blomberg’s *K* (Blomberg et al., 2003) for the number of flowers (0.12). This parameter was not calculated for AA concentration, since we only had one sample in most cases (Supplementary Material 4).

## Discussion

Most of the previously described large-scale phylogenetic relationships in *Fritillaria* were also found in our study (Rønsted et al., 2005; Day et al., 2014). The phylogenetic tree of fritillaries, based on five sequences, resolved the genus as monophyletic, which was also obtained in previous studies. However, subtrees inferred from plastid sequence data, also as in previous studies (Rønsted et al., 2005; Day et al., 2014), do not resolve *Fritillaria* as monophyletic, with the *Lilium* species nested within the studied genus.

The diversity of flower traits and pollination systems observed in fritillaries is likely due to several changes in the ecology and evolution of the studied genus. The most recent common ancestors of *Fritillaria* probably had purple or pink flowers of medium size, with the reproductive elements arranged in a similar manner to those of modern species. The ancestor likely had flowers with medium amounts of nectar rich in sugars, but with lower AA amount compared to modern species. We did not find phylogenetic signal for most of the studied traits, which indicate lack of tendency for closely related *Fritillaria* species to resemble one another. This result may suggest that several important shifts in floral characteristics e.g., flower colour in *Fritillaria* may be related to plant–pollinator interactions or other habitat-related factors. We were especially curious to study the new flower traits, e.g., reward properties in the context of new pollinators preferences. Our study revealed, for example, that flower colour is particularly good at separating bird- from insect-pollinated flowers. Colour shift may have been one of the triggers for bird pollination in *Fritillaria*. Red colour appeared among the modern, hummingbird-pollinated members of the subgenus *Rhinopetalum*. Similarly, in Asian species pollinated by passerine birds, orange flowers appeared. Red and orange flowers in *Fritillaria* are only found among bird-pollinated species, and in contrast to studies on *Iochroma* (Smith et al., 2007), *Iris* (Roguz et al., 2020a), and *Polemonium* (Landis et al., 2018), there seems to be a clear correlation between pollinator type and flower colour in the case of ornithophilous *Fritillaria.*

The two most common colour shifts were purple/green and purple/yellow, in both directions. We assume that these transitions types may have had little or no association with the influence of interacting animals. Green-coloured floral parts, common among *Fritillaria* species and most probably not visually attractive for pollinators (Roguz et al., 2020b), may be related to plant physiology. The photosynthetic activity of green flowers may be sufficient to maintain their own structures (Bazzaz and Carlson, 1979). Additionally, transition to a purple, anthocyanin-based colour, can result from environmental stress. Increased anthocyanin production may correlate with the level of sun damage or protection against herbivory (Chalker-Scott, 1999; Winkel-Shirley, 2002; Arista et al., 2013; Irwin et al., 2013). However, purple colouration may reduce visual attractiveness for insect pollinators, since several bee species have preference toward yellow flowers (Lunau and Maier, 1995; Spaethe et al., 2001). In some *Fritillaria* species, such as *F. persica*, both colour morphs (green and purple) are maintained, which is not typical. In most cases, one of the colour morphs will eventually be lost and the species will again be monomorphic (Ellis and Field, 2016). While gene flow usually leads to the fixation of one colour morph over the long term (Gray and McKinnon, 2007), some factors, including environmental heterogeneity (Tucić et al., 1998) and vegetative reproduction by rhizomes, may also maintain colour polymorphism in *Fritillaria*.

Tracking flower colour shifts in fritillaries suggests that colour loss in this genus may be reversible, in contrast to studies of other plant families (Smith and Goldberg, 2015; Landis et al., 2018). For example, the common ancestor of *F. eduardii* and *F. sewerzowii* most likely had orange flowers, while *F. sewerzowii* regains the purple colour present in the deeper nodes. The exception to this rule seems to be white flower colouration, which, similarly to *Iris*, is probably irreversible (Roguz et al., 2020a).

Other flower traits may also be related to plant–pollinator interactions in *Fritillaria*. Strong corolla constriction found in American hummingbird-pollinated fritillaries, and anthers and stigmas exerted beyond the corolla may promote contact between the birds’ bodies and the flower reproductive parts (Temeles et al., 2006; Martén-Rodríguez et al., 2009, 2010). A shift to bird pollination may also account for the increased size of the corolla entrance in the subgenus *Petilium*. These passerine bird-pollinated species have large flowers, growing close to the stem on a relatively short scape, with long stigma and anthers, distant from the tepals. These new traits may enable both to exploit the reward and make contact with anthers and stigmas. Nevertheless, similar to Dupont et al. (2004), we found no unique set of differences in the arrangement of flower reproductive parts between ornithophilous species and their entomophilous relatives. For example, long, exerted styles and stamens often associated with bird pollination (Beardsley et al., 2003), were found in both insect- and bird-pollinated *Fritillaria* species. These results may also suggest that some of the studied flower traits are just by change present in the bird-pollinated *Fritillaria* and do not result from pollinator driven trait evolution.

Flower size or arrangement of the reproductive elements often enable legitimate pollinators to access the reward. It’s properties seem to play an important role in the evolution of new pollination systems in fritillaries (Roguz et al., 2018, 2019). Our study revealed that the ability to produce AA-rich nectar with low sugar concentration evolved only once, in the case of the subgenus *Petilium*. These tendencies fulfil many of the criteria that are characteristic of passerine bird-pollinated flowers. On the other hand, the ability to produce both highly concentrated and/or copious nectar evolved several times, for example, in *F. raddeana* and *F. recurva*. The ability to produce highly concentrated nectar has never been lost, which may be related to the huge proportion of bee-pollinated fritillaries. The scarlet flowers of hummingbird pollinated species also produce reward attractive for its bird pollinators—copious amounts of relatively diluted nectar, with low concentrations of AA.

It is important to note that flower size and entrance, the arrangement of reproductive elements, or reward properties do not exclude insect pollinators in bird-pollinated fritillaries. Moreover, balanced proportions of different sugar types found in hummingbird-pollinated flowers may attract both insects and birds (Roguz et al., 2018). This scenario is also supported by the fact that *F. eastwoodiae*, which is a cross between *F. micrantha* (insect-pollinated) and *F. recurva* (bird-pollinated), must have arisen as a hybrid between two insect-visited species (Dewoody et al., 2013), since the flowers of *F. micrantha* are too small for other pollen vectors.

We also assume a pollinator shift within insect-pollinated species. In the case of fly-pollinated, typically purple-flowered *F. camtschatcensis* the reward properties (i.e., small amounts of highly concentrated nectar) and the petal structure (uneven surface, covered with numerous protrusions) may act as a trigger for pollinator shift. Flowers of this species, looking like and emitting the smell of rotting flesh, may attract new pollinators. On the other hand, the way of nectar presentation (thin film, almost solid) excludes most of the insect groups. Observed pollinator shifts in *Fritillaria* follow the predominant directionality in angiosperms, from insect to bird pollination (Wilson et al., 2007; Rausher, 2008; Van der Niet and Johnson, 2012). This transition can be explained by the more efficient transfer of pollen in bird-pollinated plants (Thomson and Wilson, 2008). However, we recorded potential for several reversals, from hummingbird- to insect-pollination, and from passerine bird- to insect-pollination. The most striking example is *F. raddeana*, a member of the subgenus *Petilium*. Flowers of this species resemble those of *F. eduardii* and *F. imperialis* but are half the size and pale-yellow-green in colour. The reward is also typical for insect-pollinated species, with a small volume of highly concentrated nectar, rich in sucrose and with low AA content (Roguz et al., 2018, 2019).

In addition to *F. raddeana*, it is likely that most of the floral diversity in *Fritillaria* is the result of adaptation to insect pollinators. Several species, even distantly related, for example *F. pudica* and *F. carica*, have similar flower traits, including colour, flower size, nectary shape (Rix and Strange, 2014), and reward properties. These similarities may reflect adaptation to pollinator preferences, especially taking into consideration species similarity and lack of phylogenetic signal. Insect-pollinated *Fritillaria* flowers are also of medium size, with anthers and stigmas that are often shorter than tepals, enabling small insects to touch the reproductive parts while foraging. Insect-pollinators often seek flowers with hexose-dominated nectar of medium volume and AA concentration, which are common in *Fritillaria* flowers (Roguz et al., 2018, 2019).

Finally, it is important to note the potential influence of other environmental factors on the evolution of *Fritillaria* flowers. Our study suggests that changes in *Fritillaria* flowers occurred rather recently, with more than 10 changes in the phenotypic optima of studied flower traits. Many flower traits were similar at the level of the deep internal nodes, whereas most changes in floral display and nectar properties appeared in shallow internal nodes. This evolutionary pattern and lack of phylogenetic signal for most traits suggest forces that act on an ecological time scale, rather than changes associated with deep phylogenetic relationships (Gómez et al., 2016). Fritillaries are found in a variety of climatic regions and in different habitats, including coasts, riparian zones, meadows, woodland, steppe, deserts, mountain screes, and alpine zones (Tomović et al., 2007; Tekñen and Aytaç, 2008; Zox and Gold, 2008; Hill, 2011, 2016; Tekñen and Aytaç, 2011; Rix and Strange, 2014; Zych et al., 2014; Gao et al., 2019). Consequently, some aspects of the floral display may have arisen by selection pressure exerted by abiotic factors related to the habitat type, such as temperature, altitudinal gradients, or water stress (Zhao and Wang, 2015; Landis et al., 2018; Gao et al., 2019). In addition, some of the observed variability should be attributed to the potential for natural hybridisation (Naruhashi et al., 2006; Kawano et al., 2008; Hill, 2011).

Further studies of *Fritillaria* flower traits and pollination systems in natural habitats, as well as molecular analyses of flower traits, would be of great importance. The results obtained in such studies may be crucial for understanding the influence of pollinators on the flower traits evolution.

## Data Availability Statement

The datasets presented in this study can be found in online repositories. The names of the repository/repositories and accession number(s) can be found below: https://www.ncbi.nlm.nih.gov/genbank/, MW081399 and https://www.ncbi.nlm.nih.gov/genbank/, MW081400.

## Author Contributions

KR and MZ conceived the study and wrote the draft version of the manuscript. KR and LH assembled field data. KR performed the nectar analysis. KR performed phylogenetic analyses. KR, MZ, AR, and LH analysed the data. All authors contributed to the final version.

## Conflict of Interest

The authors declare that the research was conducted in the absence of any commercial or financial relationships that could be construed as a potential conflict of interest.

## Footnotes

1 https://github.com/nylander/catfasta2phyml

2 www.fritillariaicones.com

3 http://www.efloras.org

4 http://www.fritillaria.org.uk

## Supplementary Material

Supplementary Figure 1 Schema of flower traits measurements (TEP, tepal length; ANT, stamen length; STY, stigma length; AT, anthers-tepals distance; AS, anthers-stigma distance; ANG, orientation of the flowers on the stem expressed as the angle between stem and the middle of the flower; SC, scape length; ENT, flower diameter (measured along the stem axis).

Supplementary Figure 2 Maximum likelihood trees inferred from analysis of **(A)** nuclear genome 18S subtree, **(B)** internal transcribed spacer ITS subtree, and plastid genomes **(C)** *matK* subtree, **(D)** *rbcL* subtree, **(E)** *rpl16* subtree. The incongruences found in trees based on plastid markers are marked with an ^∗^.

Supplementary Figure 3 Maximum-likelihood ancestral state reconstruction for number of flowers **(A)**, and the length of tepals (in mm) **(B)**, stamens (in mm) **(C)** and stigmas (in mm) **(D)**.

Supplementary Figure 4 Maximum-likelihood ancestral state reconstruction for the distance between anthers and tepals (in mm) **(A)**, and anthers and stigmas (in mm) **(B)**.

Supplementary Figure 5 Maximum-likelihood ancestral state reconstruction the angle between the stem and middle of the flower **(A)**, and length of the scape (in mm) **(B)**.

Supplementary Material 1 Table with accession numbers of the used DNA regions: (*matK*, *rpl16*, and *rbcL*), nuclear (18S) and internal transcribed spacer (ITS).

Supplementary Material 2 The description of the methods used for DNA extraction, amplification, and sequencing of *matK* regions for *F. biflora* and *F. olgae*.

Supplementary Material 3 List of used *Fritillaria* species with defined flower colours used in the colour-related analyses.

Supplementary Material 4 Supplementary Table 1 Results of trait-dependent diversification analysis Es-sim test for continuous traits for investigated *Fritillaria* species; Supplementary Table 2 The strength of the phylogenetic signal on continuous data (Blomberg’s K) for investigated *Fritillaria* species.
