# Supplementary figures and images for: Evolution of Bird and Insect Flower Traits in Fritillaria L. (Liliaceae)
Source: Front Plant Sci. 2021 Mar 31;12:656783. doi: 10.3389/fpls.2021.656783 (PMC8044542; doi:10.3389/fpls.2021.656783)

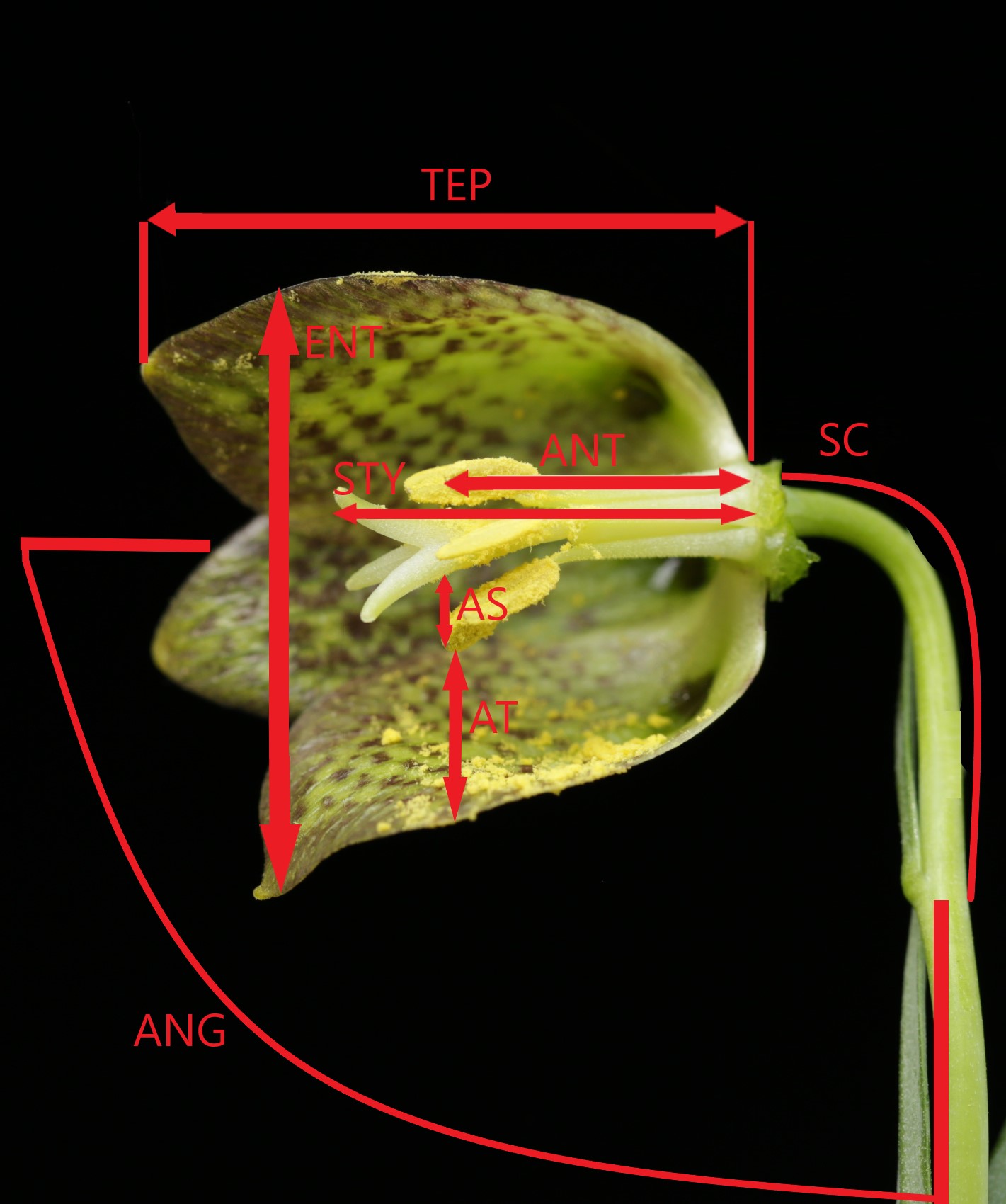

Supplement: Supplementary Figure 1 — Schema of flower traits measurements (TEP, tepal length; ANT, stamen length; STY, stigma length; AT, anthers-tepals distance; AS, anthers-stigma distance; ANG, orientation of the flowers on the stem expressed as the angle between stem and the middle of the flower; SC, scape length; ENT, flower diameter (measured along the stem axis). [file Image_1.JPEG]

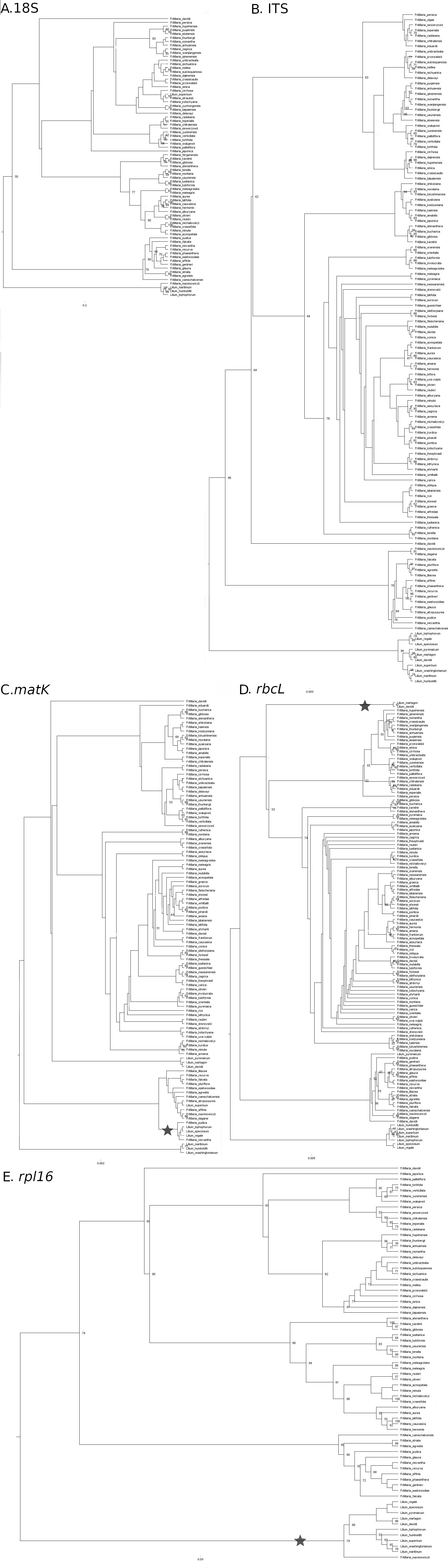

Supplement: Supplementary Figure 2 — Maximum likelihood trees inferred from analysis of (A) nuclear genome 18S subtree, (B) internal transcribed spacer ITS subtree, and plastid genomes (C) matK subtree, (D) rbcL subtree, (E) rpl16 subtree. The incongruences found in trees based on plastid markers are marked with an ∗. [file Image_2.JPEG]

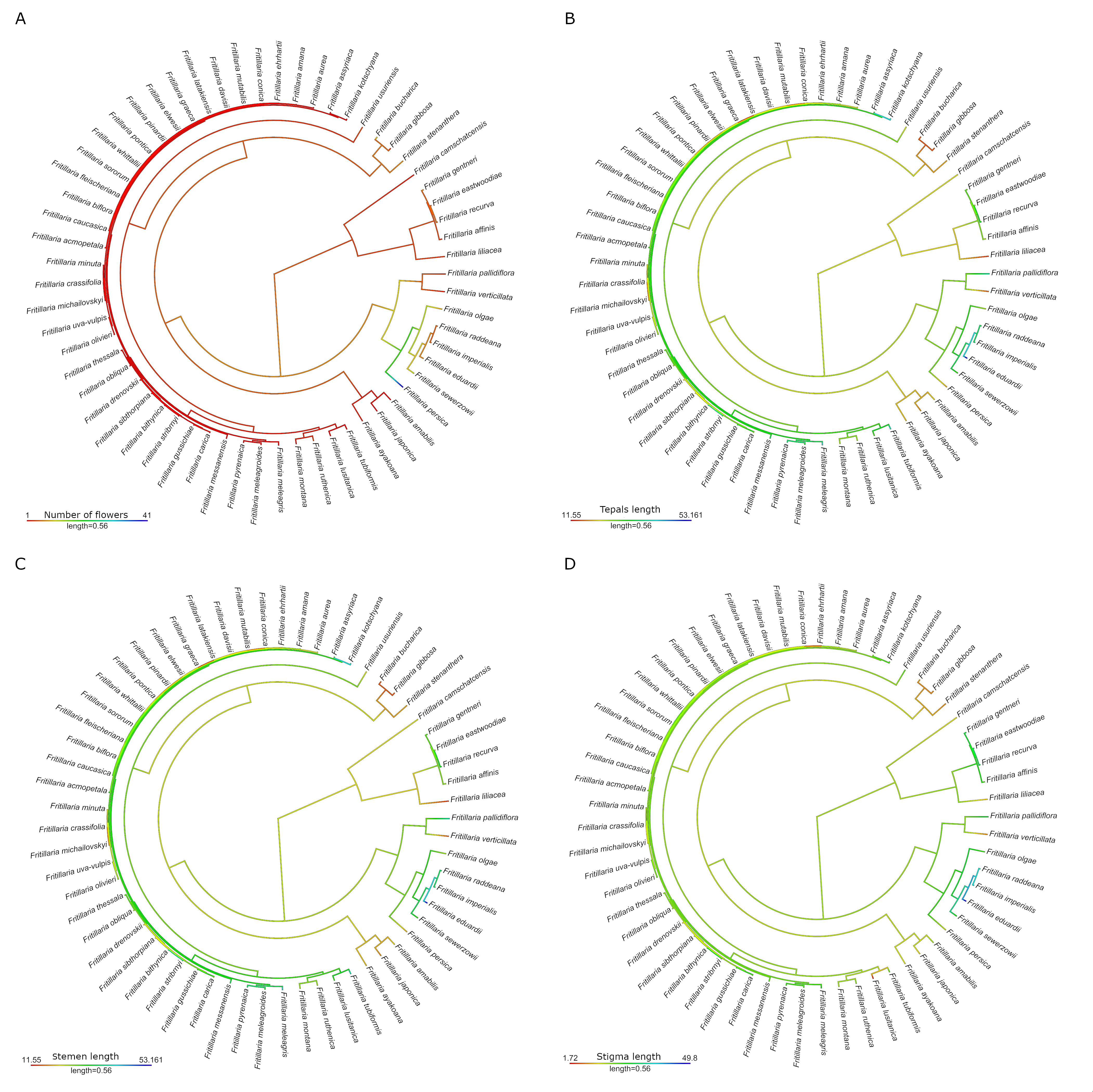

Supplement: Supplementary Figure 3 — Maximum-likelihood ancestral state reconstruction for number of flowers (A), and the length of tepals (in mm) (B), stamens (in mm) (C) and stigmas (in mm) (D). [file Image_3.JPEG]

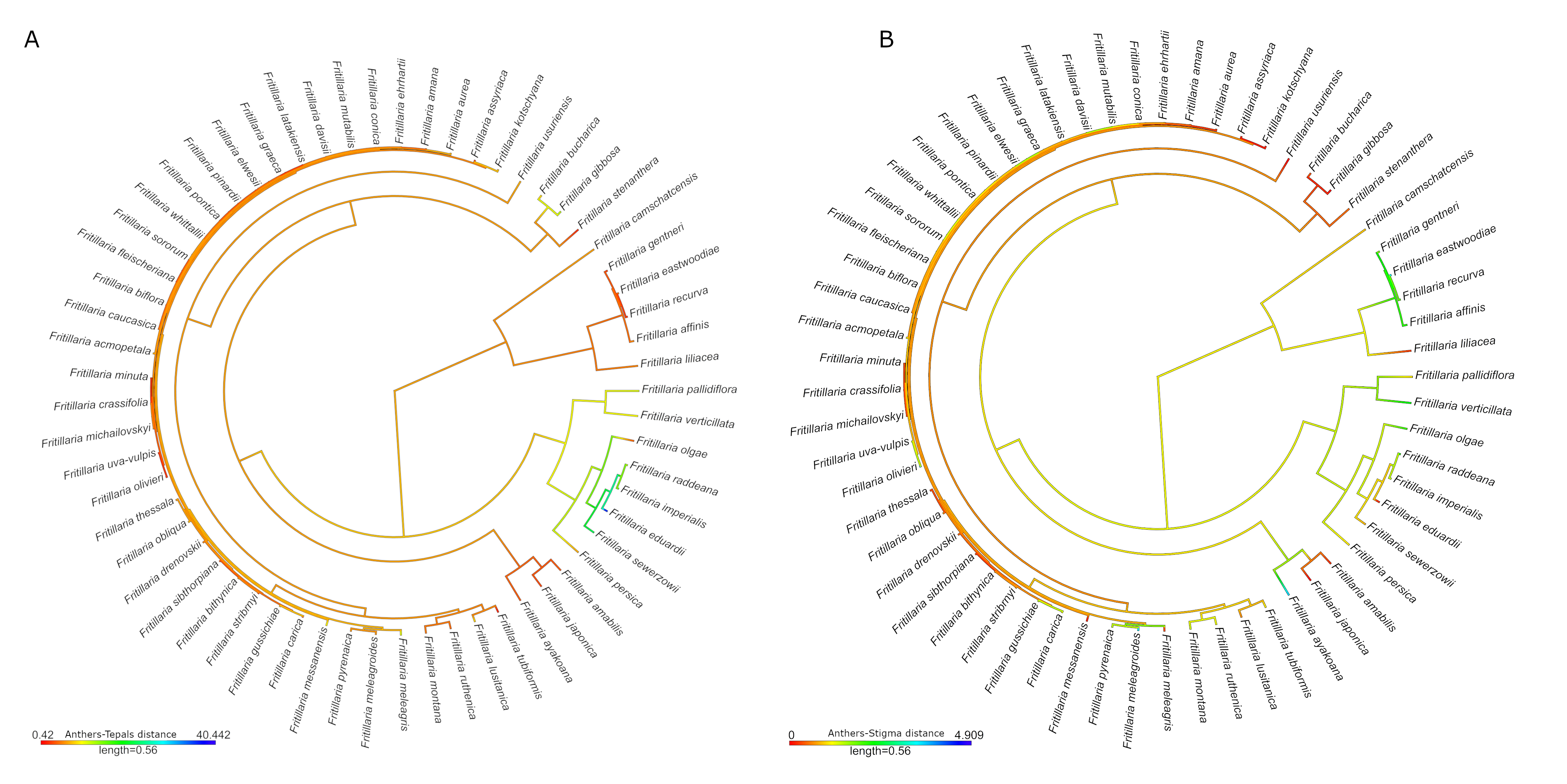

Supplement: Supplementary Figure 4 — Maximum-likelihood ancestral state reconstruction for the distance between anthers and tepals (in mm) (A), and anthers and stigmas (in mm) (B). [file Image_4.JPEG]

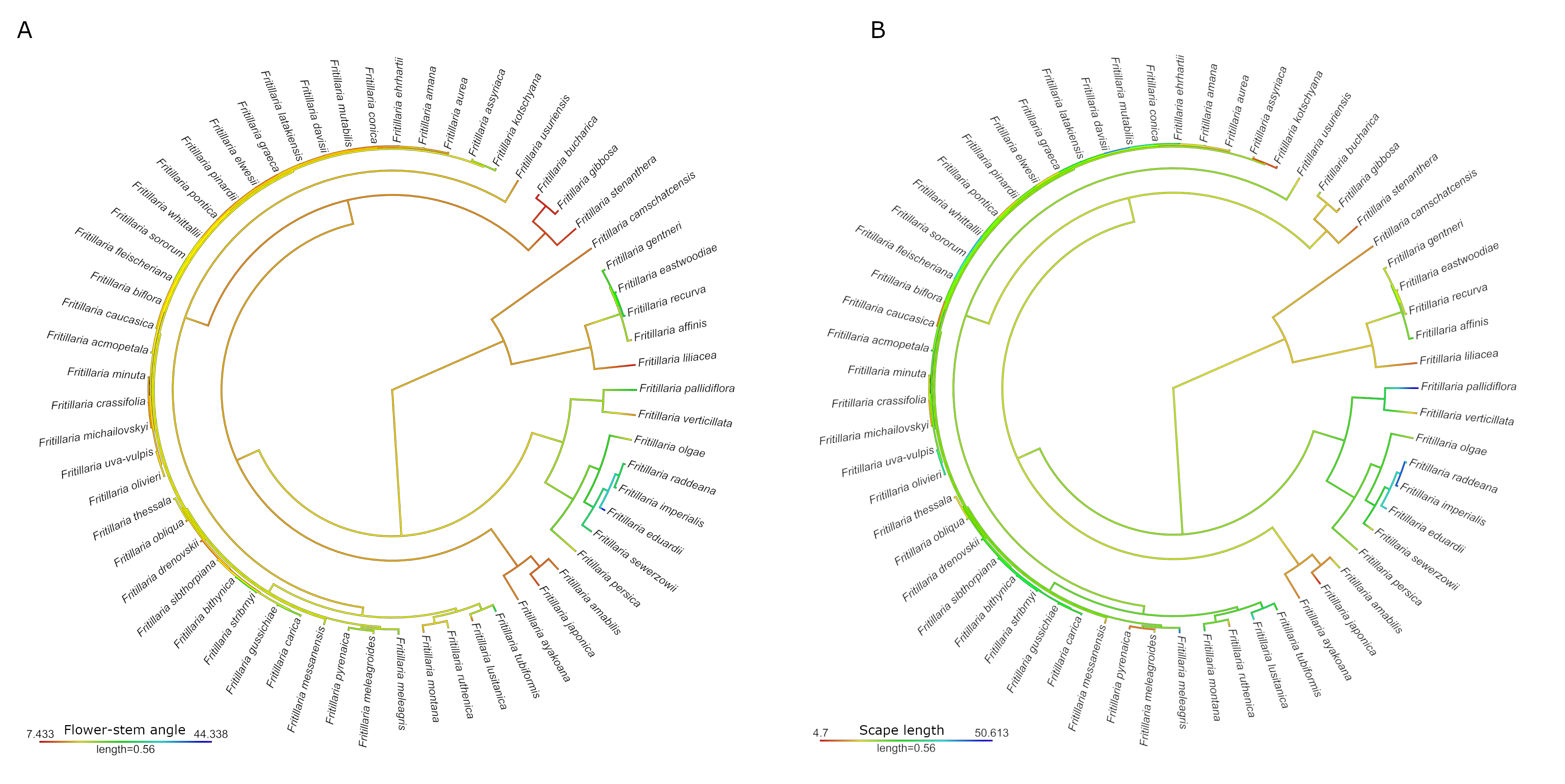

Supplement: Supplementary Figure 5 — Maximum-likelihood ancestral state reconstruction the angle between the stem and middle of the flower (A), and length of the scape (in mm) (B). [file Image_5.JPEG]
