# Supplementary material for: Evolution of Bird and Insect Flower Traits in Fritillaria L. (Liliaceae)
Source: Front Plant Sci. 2021 Mar 31;12:656783. doi: 10.3389/fpls.2021.656783 (PMC8044542; doi:10.3389/fpls.2021.656783)
Supplement: Supplementary Material 2 — The description of the methods used for DNA extraction, amplification, and sequencing of matK regions for F. biflora and F. olgae. [file Data_Sheet_2.PDF]

DNA extraction, amplification, and sequencing

For two *Fritillaria* species we sequenced the *matK* region for *F. biflora* and *F. olgae*, by extracting DNA from the living plants collection in BG. We tried to obtain also sequences of different regions, but finally due to difficulties with doing this, we obtained only *matK* regions. The samples of species used for this analysis are vouchered and deposited in a herbarium of BG. Leaf material stored in ethanol was washed 3 times in 1ml of Mili-Q water to remove excess alcohol. Leafs from silica beads were transferred to 1ml Mili-Q water for sample rehydration (15min in room temperature, occasionally mixed several times). Sample was homogenized after addition of 0,5mm glass beads (Sartorius AG, Göttingen, Germany) in a 1:1 ratio, in a tissue lyser (TissueLyser II; Qiagen, Hilden, Germany) at 20 Hz for 15 min. Obtained homogenate was used for DNA isolation using Plant DNA Isolation Kit (Eurx). matK fragment was amplified with use of the primers: -19F 5-CGT TCT GAC CAT ATT GCA CTA TG-3 and 1565R 5- TCA CCA GGT CAT TGA CAC GAA-3 (Molvray et al., 2000, Zarrei et al., 2009) PCR was done using KAPA Robust PCR kit (Roche). PCR reaction was carried out in 20 µl volume consisting of: 4 µl of KAPA 2G A buffer, 0.4 µl of 10 mM dNTPs, 1U of KAPA Robust polymeraseFLIVE, 0.5 µl of each primer (10 µM), 11.45 µl of PCR-grade water and 2 µl of DNA template. Amplification reaction conditions were as follows: 3 min of initial denaturation at 95°C, followed by 35 cycles of 30 s at 95°C, 20 s at 50°C, 1 min at 72°C, and final extension period of 2 min at 72°C. The amplified products were visualized by agarose gel electrophoresis (1.0%, wt/v) and ethidium bromide staining. Obtained PCR products were purified using EPPiC Fast kit (A&A Biotechnology, Gdańsk, Poland) and directly sequenced using BigDye Terminator v3.1 (Thermo, Life Technologies) chemistry kit on ABI3730XL genetic analyzer (Thermo, Life Technologies) in the DNA Sequencing and Oligonucleotide Synthesis Laboratory IBB PAS.
