# Supplementary material for: Evolution of Bird and Insect Flower Traits in Fritillaria L. (Liliaceae)
Source: Front Plant Sci. 2021 Mar 31;12:656783. doi: 10.3389/fpls.2021.656783 (PMC8044542; doi:10.3389/fpls.2021.656783)
Supplement: Supplementary Material 4 — Supplementary Table 1 Results of trait-dependent diversification analysis Es-sim test for continuous traits for investigated Fritillaria species; Supplementary Table 2 The strength of the phylogenetic signal on continuous data (Blomberg’s K) for investigated Fritillaria species. [file Data_Sheet_4.docx]

Table 1. Results of Es-sim test for continuous traits for investigated *Fritillaria* species*.* Studied flower traits: ENT – entrance diameter, SC – scape length, TEP – tepal length, ANT – stamen length, AS – anthers-style distance, AT – anthers-tepals distance, STY – style length, NB – number of flowers in the inflorescence, ANG – angle between the stem and the middle of the flower, V – nectar volume, CON – nectar sugar concentration, MAS – nectar mas.

|  | ENT | SC | TEP | ANT | AS | AT | STY | NB | ANG | V | CON | MAS | AA |
| --- | --- | --- | --- | --- | --- | --- | --- | --- | --- | --- | --- | --- | --- |
| Pearson's correlation coefficient | 0.17 | 0.21 | 0.14 | 0.22 | 0.14 | 0.10 | 0.34 | -0.18 | -0.09 | 0.26 | -0.31 | 0.24 | 0.18 |
| p-value | 0.44 | 0.33 | 0.50 | 0.28 | 0.51 | 0.63 | 0.09 | 0.37 | 0.67 | 0.15 | 0.10 | 0.23 | 0.39 |

Table 2. Values of Blomberg’s *K* for investigated *Fritillaria* species*.* Studied flower traits: ENT – entrance diameter, SC – scape length, TEP – tepal length, ANT – stamen length, AS – anthers-style distance, AT – anthers-tepals distance, STY – style length, NB – number of flowers in the inflorescence, ANG – angle between the stem and the middle of the flower, V – nectar volume, CON – nectar sugar concentration, MAS – nectar mas.

|  | ENT | SC | TEP | ANT | AS | AT | STY | NB | ANG | V | CON | MAS |
| --- | --- | --- | --- | --- | --- | --- | --- | --- | --- | --- | --- | --- |
| K | 0.00 | 0.00 | 0.00 | 0.00 | 0.00 | 0.00 | 0.00 | 0.12 | 0.00 | 0.02 | 0.00 | 0.01 |
| p-value | 0.02 | 0.67 | 0.64 | 0.14 | 0.37 | 0.12 | 0.23 | 0.00 | 0.38 | 0.12 | 0.39 | 0.33 |
